# Prediction of 60-day case fatality after aneurysmal subarachnoid hemorrhage: external validation of a prediction model

**DOI:** 10.1186/cc14547

**Published:** 2015-03-16

**Authors:** S Dijkland, B Roozenbeek, P Brouwer, H Lingsma, D Dippel, L Vergouw, M Vergouwen, M Van der Jagt

**Affiliations:** 1 Erasmus MC - University Medical Center, Rotterdam, the Netherlands

## Introduction

Aneurysmal subarachnoid hemorrhage (SAH) is a devastating disease with substantial morbidity and mortality. Prognostic modeling is an important instrument to identify high-risk patients in both clinical practice and research settings. Recently, a prognostic model to predict 60-day case fatality after aneurysmal SAH was developed with data from the International Subarachnoid Aneurysm Trial (ISAT) [1]. Our aim was to externally validate this model in a retrospective cohort of consecutive SAH patients.

## Methods

We included consecutive aneurysmal SAH patients admitted to one university hospital between October 2007 and October 2011. Exclusion criteria were: age <18 years, hospital admission >28 days after SAH, nonaneurysmal SAH, explicit objection by the patient to view the medical data and missing data on 60-day case fatality. The model's predictors were age, maximum lumen size of the aneurysm, Fisher grade and World Federation of Neurological Surgeons (WFNS) grade. Two versions of the model were validated: one with WFNS grade scored on admission and the other with WFNS grade assessed at the time of treatment decision, as a proxy to WFNS grade at randomization used in the ISAT. The outcome was 60-day case fatality. Model performance was assessed by studying discrimination, expressed by the area under the receiver operating characteristic curve (AUC), and calibration.

## Results

A total of 307 patients were included in the validation cohort. The observed 60-day case fatality rate was 30.6%. Discrimination was good, and was considerably better for the model with WFNS grade at treatment decision (AUC = 0.89) compared with the model with WFNS grade on admission (AUC = 0.82). Calibration was poor, with mean predicted probabilities of 17.0% for the model with WFNS grade on admission and 17.7% for the model with WFNS grade at the time of treatment decision.

## Conclusion

Our results indicate that the ISAT prediction model is generalizable, since the model showed adequate performance in an independent, unselected cohort of aneurysmal SAH patients. The model discriminated well between patients who died and those who survived the first 60 days after SAH. Additionally, use of WFNS grade at the time of treatment decision of the ruptured aneurysm improved model performance. However, since predicted probabilities were lower than observed probabilities, the ISAT prediction model needs to be adapted before use in clinical practice.
